# Acid ceramidase and its inhibitors: a *de novo* drug target and a new class of drugs for killing glioblastoma cancer stem cells with high efficiency

**DOI:** 10.18632/oncotarget.22637

**Published:** 2017-11-07

**Authors:** Ninh B. Doan, Hisham Alhajala, Mona M. Al-Gizawiy, Wade M. Mueller, Scott D. Rand, Jennifer M. Connelly, Elizabeth J. Cochran, Christopher R. Chitambar, Paul Clark, John Kuo, Kathleen M. Schmainda, Shama P. Mirza

**Affiliations:** ^1^ Biotechnology and Bioengineering Center, Medical College of Wisconsin, Milwaukee, Wisconsin, 53226, USA; ^2^ Department of Neurosurgery, Medical College of Wisconsin, Milwaukee, Wisconsin, 53226, USA; ^3^ Medicine, Hematology/Oncology, Medical College of Wisconsin, Milwaukee, Wisconsin, 53226, USA; ^4^ Radiology, Medical College of Wisconsin, Milwaukee, Wisconsin, 53226, USA; ^5^ Neurology, Medical College of Wisconsin, Milwaukee, Wisconsin, 53226, USA; ^6^ Pathology, Medical College of Wisconsin, Milwaukee, Wisconsin, 53226, USA; ^7^ Department of Neurological Surgery and Human Oncology, University of Wisconsin, Madison, Wisconsin, 53792, USA; ^8^ Biophysics, Medical College of Wisconsin, Milwaukee, Wisconsin, 53226, USA; ^9^ Obstetrics and Gynecology, Medical College of Wisconsin, Milwaukee, Wisconsin, 53226, USA; ^10^ Department of Chemistry and Biochemistry, University of Wisconsin, Milwaukee, Wisconsin, 53211, USA

**Keywords:** glioblastoma, acid ceramidase, carmofur, glioblastoma cancer stem cells (GSCs), overall survival

## Abstract

Glioblastoma remains the most common, malignant primary cancer of the central nervous system with a low life expectancy and an overall survival of less than 1.5 years. The treatment options are limited and there is no cure. Moreover, almost all patients develop recurrent tumors, which typically are more aggressive. Therapeutically resistant glioblastoma or glioblastoma stem-like cells (GSCs) are hypothesized to cause this inevitable recurrence. Identifying prognostic biomarkers of glioblastoma will potentially advance knowledge about glioblastoma tumorigenesis and enable discovery of more effective therapies. Proteomic analysis of more than 600 glioblastoma-specific proteins revealed, for the first time, that expression of acid ceramidase (ASAH1) is associated with poor glioblastoma survival. CD133+ GSCs express significantly higher ASAH1 compared to CD133- GSCs and serum-cultured glioblastoma cell lines, such as U87MG. These findings implicate ASAH1 as a plausible independent prognostic marker, providing a target for a therapy tailored toward GSCs. We further demonstrate that ASAH1 inhibition increases cellular ceramide level and induces apoptosis. Strikingly, U87MG cells, and three different patient-derived glioblastoma stem-like cancer cell lines were efficiently killed, through apoptosis, by three different known ASAH1 inhibitors with IC50's ranging from 11–104 μM. In comparison, the standard glioblastoma chemotherapy agent, temozolomide, had minimal GSC-targeted effects at comparable or even higher concentrations (IC50 > 750 μM against GSCs). ASAH1 is identified as a *de novo* glioblastoma drug target, and ASAH1 inhibitors, such as carmofur, are shown to be highly effective and to specifically target glioblastoma GSCs. Carmofur is an ASAH1 inhibitor that crosses the blood-brain barrier, a major bottleneck in glioblastoma treatment. It has been approved in Japan since 1981 for colorectal cancer therapy. Therefore, it is poised for repurposing and translation to glioblastoma clinical trials.

## INTRODUCTION

Glioblastoma remains the most common, malignant primary cancer of the central nervous system despite many decades of research [1]. Life expectancy is less than two years with the best medical and surgical treatments [2]. The current standard includes maximally safe resection, followed by combined radiation therapy and chemotherapy with temozolomide [2, 3]. Even with an initial successful treatment, glioblastoma inevitably recurs, and most patients do not survive for more than one year after recurrence [4, 5]. Bevacizumab, an angiogenesis inhibitor, has been approved for the treatment of recurrent glioblastoma [6]. Even with the addition of bevacizumab treatment, survival is not significantly improved [7] and recently, bevacizumab has been shown to have almost no effect on overall survival in newly diagnosed glioblastoma [8, 9].

A potential new chemotherapeutic target for these tumors, called acid ceramidase, has been described [10]. ASAH1, a lysosome cysteine amidase, plays an important role in the metabolism of sphingolipids [11]. ASAH1 converts ceramide into sphingosine and free fatty acid [12–14]. Sphingosine is phosphorylated into sphingosine-1-phosphate (S1P) by sphingosine kinase 1 (SPHK1) or 2 (SPHK2). Ceramide has been shown to induce apoptosis whereas sphingosine-1-phosphate functions as a tumor promotor [12–14]. Cell death induced by radiotherapy, chemotherapy, or proapoptotic ligands are mediated by ceramide [15]. Neural stem cell differentiation is mediated via direct binding to the kinase PKCz by ceramide [15]. S1P has been shown to stimulate glioblastoma cell invasiveness *in vitro* by up-regulation of the urokinase plasminogen activator, its receptor, and proinvasive molecule CCN1 [16, 17]. ASAH1 has been shown to play a significant role in tumor progression in many cancers, including melanoma, colon, and prostate cancers [18–20]. Consequently, multiple studies have suggested ASAH1 as a novel anticancer drug target [11, 21]. However, none has implicated ASAH1 to play a significant role in the cancer biology of glioblastoma.

Recent findings have also suggested that glioblastoma stem-like cells (GSCs) may play a significant role in the resistance of cancer to chemotherapy and radiotherapy [22, 23]. The cell membrane marker CD133 has been identified as a GSC marker [24, 25]. Higher expression levels of CD133 are associated with poorer prognosis [24]. Patient-derived GSCs have been isolated and are highly efficient at xenograft formation when implanted into brains of immunodeficient mice [26]. However, depletion of GSCs prior to implantation markedly reduces tumor formation [27].

The lack of effective treatment for glioblastoma, together with the recent findings regarding the role of GSCs, has generated intense interest in developing new biomarkers and GSC-targeted therapies to reduce tumor recurrence and improve patient survival. Mass-spectrometry (MS)-based proteomics analysis is emerging as a viable, high throughput method for discovering disease biomarkers by simultaneous, efficient quantitative analysis of many targets. Recent optimization of this method by us for analyzing protein markers in glioblastoma has been developed using banked human glioblastoma specimens associated with clinical parameters and outcome data from our institutional Brain and Spinal Cord Tissue Bank [28]. Using this approach we identified 601 proteins to be differentially expressed in glioblastoma [28]. In this study, we quantitated their correlation with survival by linear regression.

Here, we report that ASAH1, having the best correlation with survival of all studied proteins, is negatively correlated with glioblastoma survival. A higher expression level of ASAH1 was seen in patients with worse overall survival. Our results also showed that CD133^+^ GSCs express a very high level of ASAH1 compared to CD133^-^ GSCs and non-stem cancer cells, such as U87MG cells. These findings implicate ASAH1 as a plausible independent prognostic maker. ASAH1 inhibitors are highly more potent than temozolomide in killing GSCs and U87MG cells. Due to its high level of expression in GSCs, ASAH1 inhibition is proposed as a new anti-glioblastoma therapy that specifically targets GSCs.

## RESULTS

### Higher expression of ASAH1 is associated with worse glioblastoma survival

**Figure 1:**
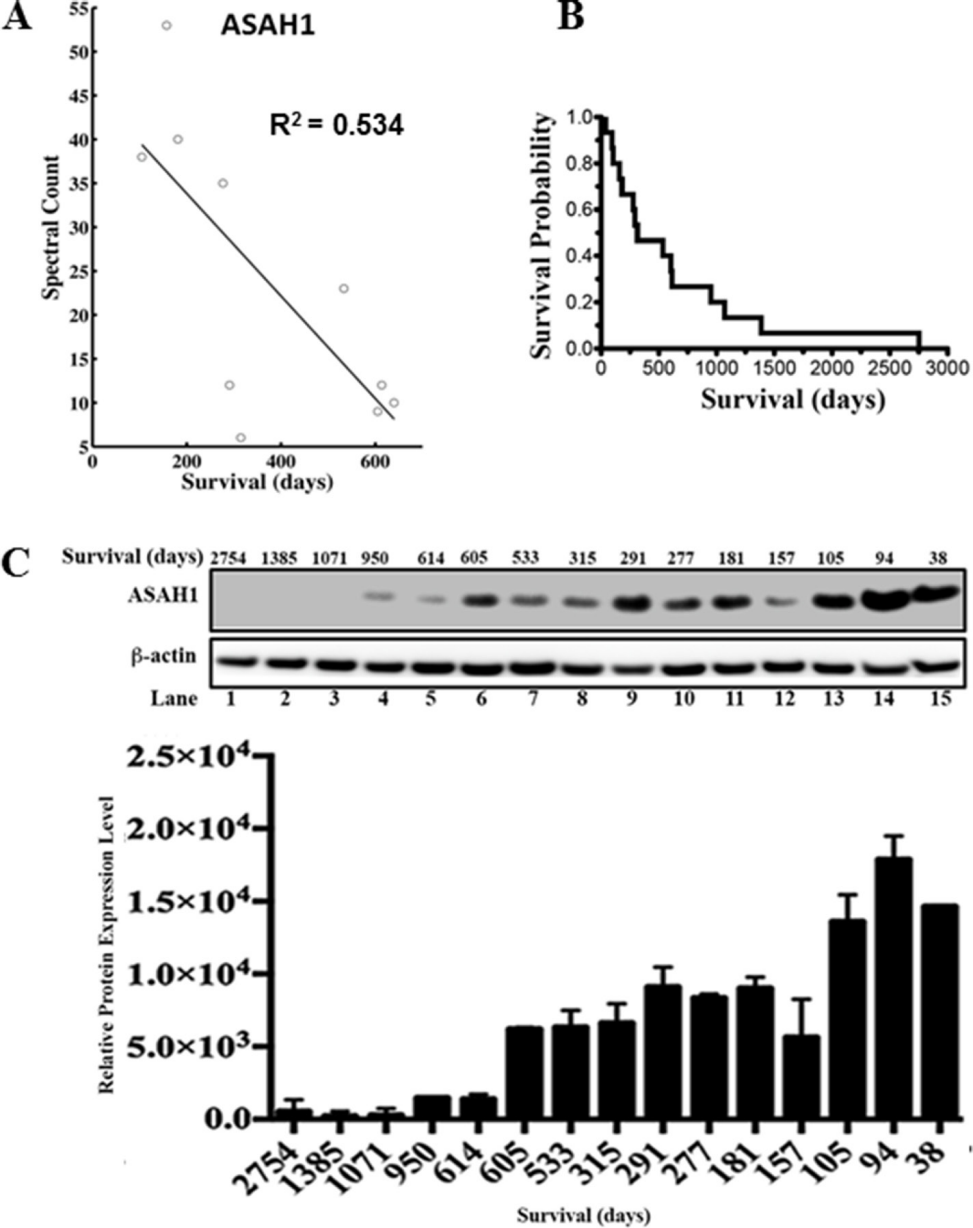
ASAH1 expression level is negatively correlated with survival (**A**) The graph of spectral count of ASAH1 vs survival is shown with data points indicated by circles. The dark line represents linear regression analysis. The coefficient of determination (R^2^) is 0.534. A negative correlation is seen between mass spectral count of ASAH1 and patient overall survival in days. (**B**) The Kaplan-Meier survival curve of 15 patients is shown. The median survival is 315 days. (**C**) Upward trending of ASAH1 expression level is seen when comparing patients with high to low overall survival. Western blot of ASAH1 and loading control β-actin confirms the negative correlation between ASAH1 and survival. Patient characteristics are shown above each lane. Quantitation of Western blot of ASAH1 with ImageJ is displayed as the graph of relative expression level vs survival in days (*n* = 3; *p* < 0.05).

Tumor tissues from 10 glioblastoma patients with known survival data were studied. A total of 601 biomarkers were identified in our previous study using the MS-based label-free quantitative proteomics by spectral counting approach [28]. In spectral counting quantification, the protein abundance is measured based on the number of MS spectra assigned to a protein. We used this mass spectral count data of the 601 proteins and plotted them against the patient overall survival data. Biomarkers were ranked based on R^2^ value, ranging from 0 to 0.53 (see Table 1 [with R^2^ value 0.2 and above]), and Supplementary Table 1 for a complete list). ASAH1 stands out with the highest R^2^ value of 0.53 among the biomarkers studied (Table 1 and Figure 1A). The correlation between protein levels and survival was evaluated by graphing mass spectral count, which correlates with protein level, against survival (see Supplementary Table 1 for fitted data of all the proteins studied). The graph of the Kaplan-Meier survival curve using our patients’ data revealed a median survival of 315 days (Figure 1B). Overall survival is higher in patients with tumors found to have a lower level of ASAH1.

**Table 1:** ASAH1 has the highest R^2^ among 601 biomarkers studied

Symbol	Name	*R*^2^
ASAH1	Acid ceramidase	5.34E-01
H90B4	Putative heat shock protein HSP 90-beta 4 HSP90AB4P	3.93E-01
STOM	Erythrocyte band 7 integral membrane protein	3.64E-01
VAT1	Synaptic vesicle membrane protein VAT-1 homolog	3.52E-01
NFH	Neurofilament heavy polypeptide NEFH	3.26E-01
PPIB	Peptidyl-prolyl cis-trans isomerase B	3.23E-01
LAMP2	Lysosome-associated membrane glycoprotein 2	3.07E-01
MYO1E	Unconventional myosin-Ie	2.95E-01
AKP13	A-kinase anchor protein 13 AKAP13	2.95E-01
HBE	Hemoglobin subunit epsilon HBE1	2.90E-01
HBD	Hemoglobin subunit delta	2.89E-01
LAMP1	Lysosome-associated membrane glycoprotein 1	2.88E-01
MOES	Moesin MSN	2.87E-01
SAMH1	SAM domain and HD domain-containing protein 1 SAMHD1	2.72E-01
MYH8	Myosin-8	2.71E-01
TIGD1	Tigger transposable element-derived protein 1	2.68E-01
HBB	Hemoglobin subunit beta	2.61E-01
AACT	Alpha-1-antichymotrypsin SERPINA3	2.56E-01
MYH7	Myosin-7	2.56E-01
LMNA	Prelamin-A/C	2.54E-01
CO4A	Complement C4-A C4A	2.49E-01
HBG2	Hemoglobin subunit gamma-2	2.45E-01
ARPC3	Actin-related protein 2/3 complex subunit 3	2.45E-01
CO4B	Complement C4-B C4B	2.40E-01
CAZA1	F-actin-capping protein subunit alpha-1 CAPZA1	2.38E-01
CO1A1	Collagen alpha-1(I) chain COL1A1	2.37E-01
FBW10	F-box/WD repeat-containing protein 10 FBXW10	2.37E-01
TM236	Transmembrane protein 236 TMEM236	2.36E-01
HBG1	Hemoglobin subunit gamma-1	2.34E-01
YJ005	Uncharacterized protein FLJ45252	2.30E-01
GNAL	Guanine nucleotide-binding protein G (olf) subunit alpha	2.29E-01
STT3A	Dolichyl-diphosphooligosaccharide--protein glycosyltransferase subunit	2.25E-01
PRDX2	Peroxiredoxin-2	2.13E-01
PCBP1	Poly (rC)-binding protein 1	2.09E-01
MD1L1	Mitotic spindle assembly checkpoint protein MAD1 MAD1L1	2.08E-01
COR1A	Coronin-1A CORO1A	2.00E-01
IGHM	Ig mu chain C region	2.00E-01

Biomarkers with coefficients of determination (*R*^2^) 0.2 and above are shown.

### Western blot analysis confirms mass spectrometry data of a higher expression level of ASAH1 in patients with worse survival

Given these promising findings, we decided to further investigation of ASAH1 by Western blot analysis. Clinical characteristics of the study subjects are summarized in Supplementary Table 2. Consistent with MS data, Western blot data demonstrated the trend of a lower expression level of ASAH1 in patients with higher survival (Figure 1C). These results were confirmed in multiple experiments (*n* = 3). While the correlation is high, there are variations. For example, the patient in lane 12, who had a relatively short survival, was found to have a relatively lower level of ASAH1 compared to patients with longer survival. Our finding of the association between the low expression level of ASAH1 and high survival is supported by a study by Hara et al., who showed ASAH1 to be a tumor promotor [29].

### Immunohistochemical analysis showed higher expression of ASAH1 in patients with poor survival

**Figure 2:**
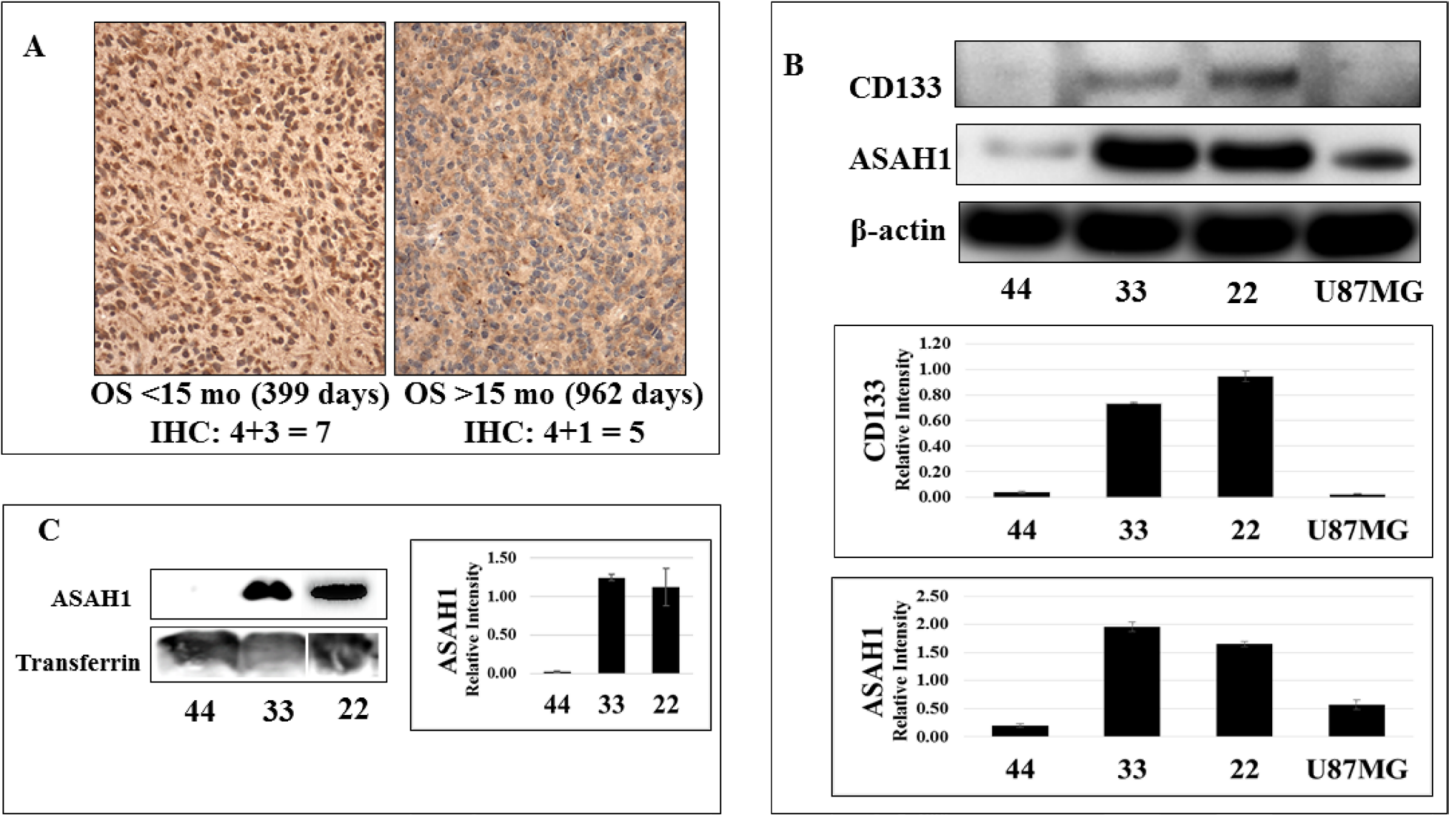
While CD133 (+) glioblastoma stem-like cancer cell lines 22 and 33 express a high level of ASAH1, limited expression of ASAH1 was seen in CD133- GSC 44 and U87MG cells (**A**) Immunohistochemical analysis of ASAH1 quantified using Allred scoring system. Left (L) represents the higher ASAH1 expression (IHC score 7) from a patient surviving < 15 months and on the right (R) the lower ASAH1 expression (IHC score 5) from a patient with longer survival > 15 months. (**B**) Western blots of GSC cell lines, 22, 33, and 44, and U87MG, cells with CD133 antibody, ASAH1 and β-actin antibodies, and (**C**) Western blots of the conditioned media of GSC lines, 22, 33, and 44 with ASAH1 and transferrin antibodies to demonstrate that ASAH1 is secreted into the media exclusively by CD133 + GSCs.

Tissue specimens from nine patients with newly diagnosed glioblastoma were subjected to immunohistochemical (IHC) staining of ASAH1. In line with Western blot assaying and MS identifications, we observed that the expression levels of ASAH1 by IHC analysis are negatively correlated with survival outcome. Quantification of the IHC staining of ASAH1 was carried out by Allred scoring system [30, 31] based on the proportion of positively stained tumor cells (proportion score, PS) and the estimated staining intensity (intensity score, IS). ASAH1 showed higher scores for specimens from short term survivors compared to the long term survivors. Figure 2A shows representative IHC images of tumor tissue from a short term survivor (< 15 months; 399 days; Left) with an IHC score of 7 (4 PS + 3 IS), and a long term survivor (> 15 months; 962 days; Right) with a score of 5 (4 PS + 1 IS), confirming the lower expression of ASAH1 in patient with longer survival. A scatter plot of IHC scores vs overall survival in days for the nine newly diagnosed GBM patient samples stained for ASAH1 is shown in supplementary data.

### CD133^+^ glioblastoma stem-like cells express high levels of ASAH1

Multiple studies have identified CD133 as a biomarker of stem-like cancer cells and their association with a poor prognosis [24]. While chemotherapy can eliminate tumor cells, it can select for or leave cancer stem cells behind, which has been proposed as a mechanism of tumor resistance [23]. Others have suggested that ASAH1 may contribute to drug resistance of tumors, perhaps by promoting and maintaining survival of cancer stem cells [22, 32, 33]. In an effort to better understand glioblastoma cancer stem cells, Zorniak et al, were able to generate and characterize several lines of patient-derived GSCs, including GSC lines 22, 33, and 44 [34]. The GSC lines 22 and 33 were identified to exhibit CD133^+^ while GSC line 44 is CD133^-^ (Figure 2B) [34, 35]. Our studies found that GSC lines 22 and 33 expressed a much higher level of ASAH1 than U87MG cells and GSC line 44, both of which are CD133^-^ (Figure 2B). ASAH1, by inhibiting apoptosis, may have played a pivotal role in promoting cell survival and proliferation of CD133^+^ GSCs, which are known to be resistant to chemo- and radiotherapy [36]. Thus, a potential therapy targeting CD133^+^ GSCs can be envisioned through the inhibition of ASAH1.

### ASAH1 is secreted into the culture media exclusively by CD133^+^ GSCs

Although ASAH1 is a non-secreted lysosomal enzyme in normal tissue, it becomes dysregulated in prostate cancer cells and is secreted into the culture media exclusively by prostate cancer cells [37]. Interestingly, we observed similar results in glioblastoma. Conditioned media of GSC lines 22, 33 and 44 were assayed with anti-ASAH1 antibody. While none or very little of ASAH1 was detected in conditioned media of the CD133^-^ GSC line 44, a significant amount of ASAH1 was detected in the conditioned medium of CD133^+^ GSC lines 22 and 33 (Figure 2C).

### ASAH1 inhibitors efficiently target U87MG and glioblastoma stem-like cells

**Figure 3:**
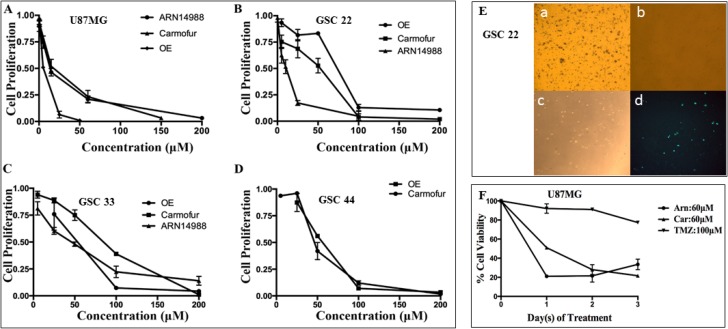
U87MG cells and GSC lines 22, 33, and 44 are highly sensitive to ASAH1 inhibitors MTT cell survival assays of (**A**) U87MG cells, (**B**) GSC 22, (**C**) GSC 33 and (**D**) GSC 44; these cells were treated with ASAH1 inhibitors, ARN14988, carmofur, and OE. (**E**) MTT assays and annexin-V fluorescent imaging of GSC line 22 a) control, b) treated with 100 μM of carmofur with ~100% cell death, c) treated with 100 μM of carmofur and fluorescently-labeled annexin-V under bright-light imaging, d) treated with 100 μM of carmofur and fluorescently-labeled annexin-V under fluorescent imaging. Carmofur kills most of the cells of GSC cell line 22. Live cells are stained blue with reduced products of MTT, as most cells in the control are, and only a few cells are alive in cells treated with carmofur. F) U87MG cells treated with three ASAH1 inhibitors from 0–3 days to demonstrate that ASAH1 inhibitors are highly cytotoxic to U87MG cells while temozolomide has a minimal effect on cell death at 72 hours. Results are expressed as mean and ± s.e.m (*N* = 3).

**Figure 4:**
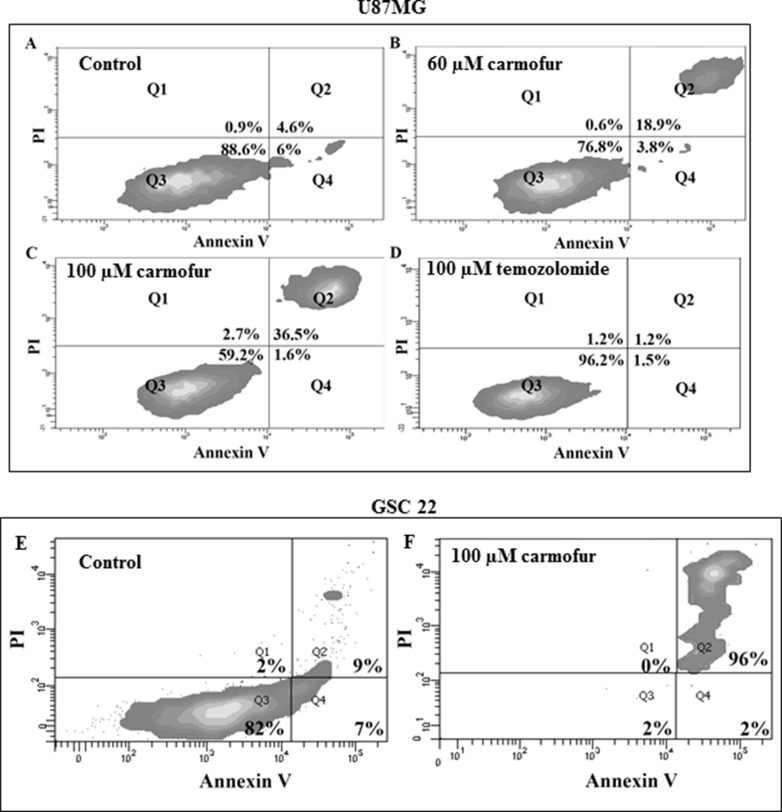
Flow cytometry studies of U87MG cells, labeling with annexin V and PI; (A) control, (B) cells treated with 60 μM carmofur, (C) cells treated with 100 μM carmofur, (D) cells treated with 100 μM temozolomide While flow cytometric experiments revealed U87MG cells treated with 60 and 100 μM of carmofur induced ~18% and 36% of cells, respectively, to undergo apoptosis, temozolomide failed to induce cell death even at a higher concentration. (**E)** and (**F**) Flow cytometry studies of GSC line 22, labeling with annexin V and PI: E) control, F) cells treated with carmofur. Approximately 96% of cells of GSC line 22 underwent apoptosis with 100 μM of carmofur, demonstrating the high efficiency of carmofur at killing glioblastoma stem-like cancer cells.

To date, no drugs have been reported to be able to induce cell death in GSCs. Temozolomide, currently the only FDA-approved oral chemotherapy of glioblastoma, is inhibiting cell growth of U87MG cells and GSCs with reported IC_50_'s of ~600 mM and 1.6 mM, respectively [3, 36, 38]. To further examine the significance of ASAH1 in survival of GSCs and U87MG cells, we used previously identified ASAH1 inhibitors, namely *N*-oleoylethanolamine (OE), carmofur, and ARN14988 [39–42]. These inhibitors were able to kill U87MG cells and three different patient-derived GSC lines, 22, 33, and 44, with IC_50_'s ranging from 11–104 mM, based on MTT assays (Figure 3, Table 2). Microscopy studies of MTT assays demonstrated that most of the cells of GSC line 22 were killed with 100 mM of carmofur as indicated by the lack of staining with reduced products of MTT assays compared to the control. These dead cells bind annexin V, indicating they were induced to undergo apoptosis by carmofur (Figure 3E). Consistent with previous studies, GSCs are more resistant to apoptosis, requiring a higher concentration of drugs to achieve a similar death rate as in non-stem-like cancer cells such as U87MG cells, which were shown to contain only ~0.15% CD133^+^ cells of the total population [36, 38, 43]. GSCs require IC_50_'s 2–7-fold higher than those of U87MG cells (Table 2). Despite a low expressing level of ASAH1 in GSC 44 cells, they remain sensitive to ASAH1 inhibitors. This may indicate that due to a different cellular environment, GSC 44 cells are also dependent on ASAH1 activity. While ASAH1 inhibitors are highly cytotoxic to both U87MG cells and GSCs, temozolomide, has minimal effects (~20%) on cell death at 72 hours at comparable or even higher concentrations (Figure 3F). Flow cytometric analysis revealed U87MG cells treated with 60 and 100 mM of carmofur for 12 hours induced ~18% and 36% of cells, respectively, to undergo apoptosis as demonstrated by the double staining with annexin V and propidium iodide (PI) (Figure 4). In contrast, temozolomide-treated U87MG cells did not undergo apoptosis as evidenced by the lack of staining with annexin V and PI (Figure 4D). These findings reveal that ASAH1 inhibitors are overwhelmingly more potent than temozolomide in killing glioblastoma cells via apoptosis. Similar findings were found with GSC line 22 when treated with carmofur. Approximately 96% of cells of GSC line 22 underwent apoptosis with 100 mM of carmofur, demonstrating the high efficiency of carmofur at killing GSCs (Figure 4E and 4F).

**Table 2:** IC50's of ASAH1 inhibitors with respect to various cell lines

Cell Lines	IC50		
	Carmofur	N-oleoylethanolamine	ARN14988
U87MG	37 μM ± 4 μM	11 μM ± 1μM	18 μM ± 4 μM
GSC 22	58 μM ± 2 μM	68 μM ± 10 μM	9 μM ± 2 μM
GSC 33	104 μM ± 7 μM	31 μM ± 3 μM	53 μM ± 10 μM
GSC 44	47 μM ± 4 μM	45 μM ± 5 μM	N.D

ASAH1 inhibitors are able to kill U87MG and glioblastoma cancer stem cells with high efficiency. Results are expressed as mean and ± S.E.M (*N* = 3). N.D, not determined, GSC, glioblastoma stem-like cell.

## DISCUSSION

Our study has identified several potential biomarkers that have not been previously characterized or known to be associated with survival in glioblastoma. This makes it difficult for further validation of our MS data due to the lack of available specific antibodies. Nonetheless, our study provides a comprehensive analysis of many biomarkers with regarding to the extent of their correlations with survival. The correlation with survival is not expected to approach the *R*^2^ value of 1, given that the tumor burden by itself is not the only factor dictating the length of survival. Patients’ comorbidities such as infection, coronary, kidney, or pulmonary diseases, etc., can significantly alter survival. Moreover, the likelihood of multiple biomarkers and pathways involved may offer further reasons for the lower correlation values.

To better understand the complexity of glioblastoma tumor biology, these biomarkers would need to be further examined regarding their roles in tumor formation and progression. Among these potential survival markers, ASAH1 demonstrated the highest correlation with survival (Table 1 and Figure 1A). These findings were confirmed with our subsequent Western blot studies, demonstrating the negative effect of ASAH1 on survival in patients with glioblastoma (Figure 1C). Consistent with our data, a study by Hara, et al. demonstrated that inhibition of ASAH1 led to an increase in apoptosis in the U87MG cell line [29]. The increase in apoptosis appears to be mediated by the increase in ceramide level, which has been shown to stimulate apoptotic cell death [12, 29]. In fact, a chemotherapy drug previously used to treat brain tumor called etoposide, a topoisomerase II inhibitor, actually mediates glial apoptosis in the C6 glioma cell line via the ceramide pathway by inducing the release of cytochrome c, and leading to the activation of caspase-9 and caspase-3 [39].

The significant role of ASAH1 in tumor progression and invasion has been well characterized in the prostate cancer cell line DU145. Investigators reported that over-expressed ASAH1 in cells resulted in larger tumor volumes that are more resistant to chemotherapy, and when ASAH1 is suppressed, cells became more sensitive to chemotherapy [20]. The growth of prostate cancer xenografts was significantly suppressed compared to conventional radiation therapy when mice were treated with B13, an ASAH1 inhibitor, and radiation, suggesting a synergistic role between these inhibitors and radiation [44].

It has been suggested that a small subpopulation of cells, the GSCs, are capable of self-renewing “stem-like” cancer cells, actually promoting tumor propagation, recurrence, and even tumor resistance to common treatment therapies [22, 27, 45, 46]. This theory implies that to be an effective anticancer therapy, it must be able to eliminate GSCs. Using U87MG cells, and GSC lines 22, 33, and 44, we found ASAH1 to be highly expressed in only CD133^+^ cells (Figure 2). Thus, we have discovered an enzymatic biomarker that presents at a high level in CD133^+^ GSCs. Consequently, targeting ASAH1 will enable the development of an anticancer therapy tailored towards GSCs. Given that the previous study has identified CD133^+^ cells as actual glioblastoma stem-like cells, we speculate that ASAH1 may decrease overall survival by enhancing survival of GSCs, and therefore, producing resistance to common anticancer therapies. In support of this hypothesis, a CD133^+^ cell subpopulation isolated from the U87MG parental cell line was shown to have increased migratory response to sphingosine 1-phosphate (S1P), an end product of ASAH1, compared to parental U87MG cells [43]. Using histologically confirmed glioma specimens, Abuhusain et al demonstrated that the shift from ceramide to S1P increases with increasing glioma cancer grade [47]. This increase in the S1P level may be due to the elevated level of ASAH1, which converts ceramide to S1P, as was demonstrated in patients with lower overall survival. This indirectly supports our finding of ASAH1 having the negative effect on survival. Patient glioblastoma tissues, in fact, contain 9-fold higher S1P and 5-fold lower ceramide concentrations compared to the normal gray matter [47]. Inhibition of S1P formation, which can be achieved by ASAH1 inhibition, also blocks angiogenesis via a pathway independent of VEGF [47]. Given these findings it can be speculated that, at least in glioblastoma, inhibition of ASAH1 achieves two critical tasks: 1) induce apoptosis and 2) block angiogenesis.

Recently, targeting ASAH1 for cancer treatments has generated significant interest, as reported by multiple studies, which identified ASAH1 as a therapeutic target. Thus, several ASAH1 inhibitors are being developed for use in different cancer treatments [21, 48]. In light of our results, we probed the role of ASAH1 in cell survival of U87MG cells and GSC lines 22, 33, and 44 using known ASAH1 inhibitors, OE, carmofur, and ARN14988. It is important to mention that carmofur was recently found to be a potent inhibitor of ASAH1 and was demonstrated to be able to cross the blood-brain barrier [41]. Carmofur has seen clinical use in Japan since 1981 for the treatment of colorectal cancers [49–51]. These inhibitors efficiently killed U87MG cells and GSCs, although with different IC_50_'s (Figure 3 and Table 2). Temozolomide, the only FDA-approved glioblastoma chemotherapy agent, has a minimal effect (~20% cell death) on U87MG cells at 72 hours compared to ASAH1 inhibitors (Figure 3F). To elucidate the mechanism of cell death induced by ASAH1 inhibitors, flow cytometric experiments were done with annexin V and PI dyes. Flow cytometric analysis revealed a significant fraction of both U87MG cells and GSC line 22 treated with carmofur underwent apoptosis as demonstrated by the double labeling of annexin V and PI (Figure 4). Consistent with MTT assays, temozolomide-treated U87MG cells did not undergo apoptosis as evidenced by the lack of staining with annexin V or PI (Figure 4D). To date, these ASAH1 inhibitors, much more effective than temozolomide, are the only reported small-molecule drugs capable of killing U87MG cells and GSCs. Thus, making them highly desired candidates for further drug development studies, or even clinical trials, given that, in Japan, carmofur has been in clinical use since1981 for the treatment of colorectal cancers [41, 48–52].

The mechanism involved in the regulation of ASAH1 may have been altered in the CD133^+^ GSCs, allowing it to be secreted into culture media (Figure 2D) or possibly into the interstitial tissues in case of an *in vivo* brain tumor. This may enable CD133^+^ GSCs to transfer their malignant potential to nearby cells. The secreted ASAH1 may, in principle, diffuse into nearby cancer cells or even normal cells, enabling them to become more resistant to radio- and chemotherapy, promoting cell growth and angiogenesis. The exclusive secretion of ASAH1 by CD133^+^ GSCs can be utilized as an advantage by developing an antibody or tumor vaccine against the secreted ASAH1, allowing a therapy specifically targeting glioblastoma.

### Implication of the study

This study has discovered both ASAH1 as a *de novo* glioblastoma drug target and a new class of drug tailored towards GSCs, given ASAH1 is highly expressed only in CD133^+^ GSCs. To our knowledge, these are the first small-molecule drugs demonstrated to be highly effective against glioblastoma stem-like cells. Among these three known inhibitors of ASAH1, carmofur, which is capable of crossing the blood-brain barrier, is already a common treatment modality for colorectal cancers in Japan since 1981. As such, patients with glioblastoma can potentially receive immediate benefits from this new class of drug.

### Experimental procedures

### Ethics statement

The research protocol was approved by the Institutional Review Board (IRB) at the Medical College of Wisconsin (MCW), Milwaukee, WI. All samples were collected after informed written consent was obtained from patients.

### Reagents and cells

Mouse antibody against ASAH1 (612302) was purchased from BD Biosciences (San Jose, CA). Anti-actin, carmofur, temozolomide, and *N*-oleoylethanolamine (OE), 3-(4,5-dimethylthiazol-2-yl)-2,5-diphenyltetrazolium bromide (MTT) were purchased from Sigma-Aldrich (St. Louis, MO). ARN14988 was a gift from Dr. Daniele Piomelli, University of California, Irvine, CA. DMSO used to dissolve carmofur, temozolomide, ARN14988, and *N*-oleoylethanolamine (OE) was purchased from Sigma Aldrich (St. Louis, MO). HRP-conjugated goat anti-mouse IgG was supplied by R&D Systems, Inc. (Minneapolis, MN). SDS-PAGE and Western blot materials and the annexin V kit were obtained from Life Technologies, Inc. (Grand Island, NY).

### Cell culture

The U87MG glioblastoma cell line was cultured in Eagle's Minimum essential medium (MEM) containing 10% (v/v) fetal bovine serum (FBS). Details of isolation and characterization of patient-derived GSC lines (22, 33, and 44) were described previously [34, 35]. Briefly, GSC lines are cultured in minimal stem cell media containing 70% DMEM-high glucose, 30% Ham's F12, 1X~ B27, 5 μg/ml heparin, 1% antibiotics, and 20 ng/ml each epidermal growth factor (EGF) and bovine fibroblast growth factor (bFGF). All cell lines were maintained in a humidified atmosphere of 5% CO_2_ at 37°C.

### Tissue collection

All samples were collected after informed written consent was obtained from patients. Briefly, glioblastoma tumor and non-tumor epilepsy tissues from consented patients were collected at the time of therapeutic tissue resection and snap frozen in liquid nitrogen within 30 minutes of removal and stored at −80°C in the Brain and Spinal Cord Tissue Bank at MCW until use. All tissues were evaluated by routine histologic, immunohistochemical and angiogenic measurements. Each tissue biopsy sample was fixed in 10% buffered formalin, processed, embedded in paraffin, cut, stained with hematoxylin and eosin and any other histochemical or immunohistochemical stains needed to fully evaluate the tissue. The diagnostic evaluation of each biopsy was performed in the Department of Pathology at MCW. Diagnosis of glioblastoma was based on morphologic features that are considered histological hallmarks of glioblastoma including high cellularity, nuclear hyperchromatism and pleomorphism, abundant mitoses, endothelial proliferation, and necrosis with or without pseudopalisades.

### Tissue homogenization

Sample preparation was performed according to the method our lab recently published [28]. Briefly, glioblastoma primary tumor samples were homogenized and powdered in liquid nitrogen using a mixer mill (Retsch Inc., Haan, Germany). Samples were maintained at liquid N_2_ temperature throughout the process. Homogenized and powdered tissue samples were then resuspended in 5X volume of the weight of the tissue sample in a reducing buffer (125 mM Tris pH 6.8, 4% SDS (w/v), 10% glycerol (v/v), 5% 2-mercaptoethanol (v/v), Complete protease inhibitor (Roche Diagnostics Corporation, Indianapolis, IN), HALT phosphatase inhibitor (Thermo Scientific, Grand Island, NY). Samples were then heated to 70°C with mixing at 1400 rpm for 10 minutes, sonicated with a tip sonicator for 30 seconds at power level 3, and then centrifuged at 16000 g for 10 minutes at room temperature. The supernatant was then collected and subjected to a Pierce 660 nm protein assay to determine protein concentration.

### Western blot analysis and quantification

Equal amounts (15 μg) of protein from each of the fifteen glioblastoma tumor samples were loaded onto the 4–12% gel. SDS-PAGE and Western blot assays were performed using standard methods [28]. 5% bovine serum albumin was used to block the membrane. A 1:500 dilution was used for primary antibody and 1:10,000 for secondary antibody. ImageJ software was used to quantify Western blot images.

### Immunohistochemical analysis

Tissue specimens from nine patients with newly diagnosed glioblastoma were subjected to immunohistochemical (IHC) staining of ASAH1. IHC staining was performed on a Dako Autostainer Plus using the Dako Envision™ FLEX High pH Detection Kit. Briefly, after deparaffinization and rehydration of the tissue, antigen retrieval was performed with Tris/EDTA pH 9. After blocking of non-target epitopes, anti-acid ceramidase primary antibody (Santa Cruz Biotechnology Inc., Dallas, TX) was applied at a concentration of 1:100 for 30 minutes, secondary antibody for 20 minutes, and DAB for 10 minutes. Hematoxylin was used as counterstain.

The IHC images were quantified to measure the ASAH1 expression level using Allred scoring system [30, 31]. Briefly, the method includes measuring the proportion score (PS), which is an estimate of the proportion of positively stained tumor cells ranging from 0–5, and the intensity score (IS), which is an estimate of the average staining intensity of the positive staining tumor cells ranging from 0–3. The sum of PS and IS result in the final IHC score for the given marker, which was then matched with the individual WHO pathology diagnoses.

### Data analysis using matlab

Matlab version R2012a was used for graphing and data fitting of results from the mass spectrometry study. The linear regression model was used to obtain the coefficient of determination (*R*^2^) from the graph of mass spectral count vs survival in days.

### 3-(4, 5-dimethylthiazol-2-yl)-2, 5-diphenyltetrazolium bromide (MTT) assays

Cells were plated at the density of 1 × 10^5^ cells/well onto a 96-well plate. Media was replaced by serum-free media after overnight incubation. Cells were treated with various drugs (OE, Carmofur, ARN14988) dissolved in DMSO for 24 hours. MTT reagents were added after 24 hours of incubation, followed by acidic-isopropanol 4 hours later to dissolve the formazan precipitate. All wells contained less than 0.2% of final concentration of DMSO. The absorbance values were recorded at wavelengths 570 and 630 nm. IC_50_'s were calculated with the GraphPad Prism software.

### Flow cytometry

Cells were plated onto a 6-well plate at the density of 1 × 10^6^ cells/ml with serum-free media. Cells were treated with carmofur and temozolomide for 12 hours then labeled with annexin V and propidium iodine (PI). A LSRII flow cytometry machine was used to record the data.

## SUPPLEMENTARY MATERIALS FIGURES AND TABLES



## References

[1] Ostrom QT, Gittleman H, Liao P, Rouse C, Chen Y, Dowling J, Wolinsky Y, Kruchko C, Barnholtz-Sloan J (2014). CBTRUS Statistical Report: Primary Brain and Central Nervous System Tumors Diagnosed in the United States in 2007–2011. Neuro Oncol.

[2] Stupp R, Mason WP, van den Bent MJ, Weller M, Fisher B, Taphoorn MJ, Belanger K, Brandes AA, Marosi C, Bogdahn U, Curschmann J, Janzer RC, Ludwin SK (2005). Radiotherapy plus concomitant and adjuvant temozolomide for glioblastoma. N Engl J Med.

[3] Lacroix M, Abi-Said D, Fourney DR, Gokaslan ZL, Shi W, DeMonte F, Lang FF, McCutcheon IE, Hassenbusch SJ, Holland E, Hess K, Michael C, Miller D, Sawaya R (2001). A multivariate analysis of 416 patients with glioblastoma multiforme: prognosis, extent of resection, and survival. Journal of neurosurgery.

[4] Ballman KV, Buckner JC, Brown PD, Giannini C, Flynn PJ, LaPlant BR, Jaeckle KA (2007). The relationship between six-month progression-free survival and 12-month overall survival end points for phase II trials in patients with glioblastoma multiforme. Neuro Oncol.

[5] Lamborn KR, Yung WK, Chang SM, Wen PY, Cloughesy TF, DeAngelis LM, Robins HI, Lieberman FS, Fine HA, Fink KL, Junck L, Abrey L, Gilbert MR (2008). Progression-free survival: an important end point in evaluating therapy for recurrent high-grade gliomas. Neuro Oncol.

[6] Chamberlain MC (2011). Bevacizumab for the treatment of recurrent glioblastoma. Clin Med Insights Oncol.

[7] Friedman HS, Prados MD, Wen PY, Mikkelsen T, Schiff D, Abrey LE, Yung WK, Paleologos N, Nicholas MK, Jensen R, Vredenburgh J, Huang J, Zheng M, Cloughesy T (2009). Bevacizumab alone and in combination with irinotecan in recurrent glioblastoma. J Clin Oncol.

[8] Gilbert MR, Dignam JJ, Armstrong TS, Wefel JS, Blumenthal DT, Vogelbaum MA, Colman H, Chakravarti A, Pugh S, Won M, Jeraj R, Brown PD, Jaeckle KA (2014). A randomized trial of bevacizumab for newly diagnosed glioblastoma. N Engl J Med.

[9] Chinot OL, Wick W, Mason W, Henriksson R, Saran F, Nishikawa R, Carpentier AF, Hoang-Xuan K, Kavan P, Cernea D, Brandes AA, Hilton M, Abrey L, Cloughesy T (2014). Bevacizumab plus radiotherapy-temozolomide for newly diagnosed glioblastoma. N Engl J Med.

[10] Doan NB, Nguyen HS, Montoure A, Al-Gizawiy MM, Mueller WM, Kurpad S, Rand SD, Connelly JM, Chitambar CR, Schmainda KM, Mirza SP (2017). Acid ceramidase is a novel drug target for pediatric brain tumors. Oncotarget.

[11] Park JH, Schuchman EH (2006). Acid ceramidase and human disease. Biochimica et biophysica acta.

[12] Pettus BJ, Chalfant CE, Hannun YA (2002). Ceramide in apoptosis: an overview and current perspectives. Biochimica et biophysica acta.

[13] Ogretmen B, Hannun YA (2004). Biologically active sphingolipids in cancer pathogenesis and treatment. Nature reviews Cancer.

[14] Taha TA, Mullen TD, Obeid LM (2006). A house divided: ceramide, sphingosine, and sphingosine-1-phosphate in programmed cell death. Biochimica et biophysica acta.

[15] Bieberich E (2008). Ceramide signaling in cancer and stem cells. Future lipidology.

[16] Young N, Van Brocklyn JR (2007). Roles of sphingosine-1-phosphate (S1P) receptors in malignant behavior of glioma cells. Differential effects of S1P2 on cell migration and invasiveness. Experimental cell research.

[17] Young N, Pearl DK, Van Brocklyn JR (2009). Sphingosine-1-phosphate regulates glioblastoma cell invasiveness through the urokinase plasminogen activator system and CCN1/Cyr61. Molecular cancer research.

[18] Pitson SM, Moretti PA, Zebol JR, Lynn HE, Xia P, Vadas MA, Wattenberg BW (2003). Activation of sphingosine kinase 1 by ERK1/2-mediated phosphorylation. The EMBO journal.

[19] Seelan RS, Qian C, Yokomizo A, Bostwick DG, Smith DI, Liu W (2000). Human acid ceramidase is overexpressed but not mutated in prostate cancer. Genes, chromosomes & cancer.

[20] Saad AF, Meacham WD, Bai A, Anelli V, Elojeimy S, Mahdy AE, Turner LS, Cheng J, Bielawska A, Bielawski J, Keane TE, Obeid LM, Hannun YA (2007). The functional effects of acid ceramidase overexpression in prostate cancer progression and resistance to chemotherapy. Cancer biology & therapy.

[21] Zeidan YH, Jenkins RW, Korman JB, Liu X, Obeid LM, Norris JS, Hannun YA (2008). Molecular targeting of acid ceramidase: implications to cancer therapy. Current drug targets.

[22] Bao S, Wu Q, McLendon RE, Hao Y, Shi Q, Hjelmeland AB, Dewhirst MW, Bigner DD, Rich JN (2006). Glioma stem cells promote radioresistance by preferential activation of the DNA damage response. Nature.

[23] Dean M, Fojo T, Bates S (2005). Tumour stem cells and drug resistance. Nature reviews Cancer.

[24] Zeppernick F, Ahmadi R, Campos B, Dictus C, Helmke BM, Becker N, Lichter P, Unterberg A, Radlwimmer B, Herold-Mende CC (2008). Stem cell marker CD133 affects clinical outcome in glioma patients. Clinical cancer research.

[25] Christensen K, Schroder HD, Kristensen BW (2008). CD133 identifies perivascular niches in grade II-IV astrocytomas. J Neurooncol.

[26] Wakimoto H, Mohapatra G, Kanai R, Curry WT, Yip S, Nitta M, Patel AP, Barnard ZR, Stemmer-Rachamimov AO, Louis DN, Martuza RL, Rabkin SD (2012). Maintenance of primary tumor phenotype and genotype in glioblastoma stem cells. Neuro Oncol.

[27] Singh SK, Hawkins C, Clarke ID, Squire JA, Bayani J, Hide T, Henkelman RM, Cusimano MD, Dirks PB (2004). Identification of human brain tumour initiating cells. Nature.

[28] Heroux MS, Chesnik MA, Halligan BD, Al-Gizawiy M, Connelly JM, Mueller WM, Rand SD, Cochran EJ, LaViolette PS, Malkin MG, Schmainda KM, Mirza SP (2014). Comprehensive characterization of glioblastoma tumor tissues for biomarker identification using mass spectrometry-based label-free quantitative proteomics. Physiological genomics.

[29] Hara S, Nakashima S, Kiyono T, Sawada M, Yoshimura S, Iwama T, Banno Y, Shinoda J, Sakai N (2004). p53-Independent ceramide formation in human glioma cells during gamma-radiation-induced apoptosis. Cell death and differentiation.

[30] Allred DC, Harvey JM, Berardo M, Clark GM (1998). Prognostic and predictive factors in breast cancer by immunohistochemical analysis. Modern pathology.

[31] Choudhury KR, Yagle KJ, Swanson PE, Krohn KA, Rajendran JG (2010). A robust automated measure of average antibody staining in immunohistochemistry images. J Histochem Cytochem.

[32] Segui B, Andrieu-Abadie N, Jaffrezou JP, Benoist H, Levade T (2006). Sphingolipids as modulators of cancer cell death: potential therapeutic targets. Biochimica et biophysica acta.

[33] Prinetti A, Millimaggi D, D’Ascenzo S, Clarkson M, Bettiga A, Chigorno V, Sonnino S, Pavan A, Dolo V (2006). Lack of ceramide generation and altered sphingolipid composition are associated with drug resistance in human ovarian carcinoma cells. The Biochemical journal.

[34] Zorniak M, Clark PA, Leeper HE, Tipping MD, Francis DM, Kozak KR, Salamat MS, Kuo JS (2012). Differential expression of 2’,3’-cyclic-nucleotide 3’-phosphodiesterase and neural lineage markers correlate with glioblastoma xenograft infiltration and patient survival. Clinical cancer research.

[35] Clark PA, Iida M, Treisman DM, Kalluri H, Ezhilan S, Zorniak M, Wheeler DL, Kuo JS (2012). Activation of multiple ERBB family receptors mediates glioblastoma cancer stem-like cell resistance to EGFR-targeted inhibition. Neoplasia.

[36] Qiu ZK, Shen D, Chen YS, Yang QY, Guo CC, Feng BH, Chen ZP (2014). Enhanced MGMT expression contributes to temozolomide resistance in glioma stem-like cells. Chinese journal of cancer.

[37] Johnson IR, Parkinson-Lawrence EJ, Butler LM, Brooks DA (2014). Prostate cell lines as models for biomarker discovery: performance of current markers and the search for new biomarkers. The Prostate.

[38] Farace C, Oliver JA, Melguizo C, Alvarez P, Bandiera P, Rama AR, Malaguarnera G, Ortiz R, Madeddu R, Prados J (2015). Microenvironmental Modulation of Decorin and Lumican in Temozolomide-Resistant Glioblastoma and Neuroblastoma Cancer Stem-Like Cells. PloS one.

[39] Sawada M, Nakashima S, Banno Y, Yamakawa H, Hayashi K, Takenaka K, Nishimura Y, Sakai N, Nozawa Y (2000). Ordering of ceramide formation, caspase activation, and Bax/Bcl-2 expression during etoposide-induced apoptosis in C6 glioma cells. Cell death and differentiation.

[40] Strelow A, Bernardo K, Adam-Klages S, Linke T, Sandhoff K, Kronke M, Adam D (2000). Overexpression of acid ceramidase protects from tumor necrosis factor-induced cell death. The Journal of experimental medicine.

[41] Realini N, Solorzano C, Pagliuca C, Pizzirani D, Armirotti A, Luciani R, Costi MP, Bandiera T, Piomelli D (2013). Discovery of highly potent acid ceramidase inhibitors with *in vitro* tumor chemosensitizing activity. Scientific reports.

[42] Realini N, Palese F, Pizzirani D, Pontis S, Basit A, Bach A, Ganesan A, Piomelli D (2015). Acid Ceramidase in Melanoma: Expression, Localization and Effects of Pharmacological Inhibition. The Journal of biological chemistry.

[43] Annabi B, Lachambre MP, Plouffe K, Sartelet H, Beliveau R (2009). Modulation of invasive properties of CD133+ glioblastoma stem cells: a role for MT1-MMP in bioactive lysophospholipid signaling. Molecular carcinogenesis.

[44] Samsel L, Zaidel G, Drumgoole HM, Jelovac D, Drachenberg C, Rhee JG, Brodie AM, Bielawska A, Smyth MJ (2004). The ceramide analog, B13, induces apoptosis in prostate cancer cell lines and inhibits tumor growth in prostate cancer xenografts. The Prostate.

[45] Clarke MF, Dick JE, Dirks PB, Eaves CJ, Jamieson CH, Jones DL, Visvader J, Weissman IL, Wahl GM (2006). Cancer stem cells--perspectives on current status and future directions: AACR Workshop on cancer stem cells. Cancer Res.

[46] Liu G, Yuan X, Zeng Z, Tunici P, Ng H, Abdulkadir IR, Lu L, Irvin D, Black KL, Yu JS (2006). Analysis of gene expression and chemoresistance of CD133+ cancer stem cells in glioblastoma. Molecular cancer.

[47] Abuhusain HJ, Matin A, Qiao Q, Shen H, Kain N, Day BW, Stringer BW, Daniels B, Laaksonen MA, Teo C, McDonald KL, Don AS (2013). A metabolic shift favoring sphingosine 1-phosphate at the expense of ceramide controls glioblastoma angiogenesis. The Journal of biological chemistry.

[48] Pizzirani D, Pagliuca C, Realini N, Branduardi D, Bottegoni G, Mor M, Bertozzi F, Scarpelli R, Piomelli D, Bandiera T (2013). Discovery of a new class of highly potent inhibitors of acid ceramidase: synthesis and structure-activity relationship (SAR). Journal of medicinal chemistry.

[49] Kubota T, Fujita S, Kodaira S, Yamamoto T, Josui K, Arisawa Y, Suto A, Ishibiki K, Abe O, Mabuchi K, Fuse M (1991). Antitumor activity of fluoropyrimidines and thymidylate synthetase inhibition. Japanese journal of cancer research.

[50] Watanabe M, Kodaira S, Takahashi T, Tominaga T, Hojo K, Kato T, Kunitomo K, Isomoto H, Ohashi Y, Yasutomi M (2006). Randomized trial of the efficacy of adjuvant chemotherapy for colon cancer with combination therapy incorporating the oral pyrimidine 1-hexylcarbamoyl-5-fluorouracil. Langenbeck's archives of surgery.

[51] Sato S, Ueyama T, Fukui H, Miyazaki K, Kuwano M (1999). [Anti-tumor effects of carmofur on human 5-FU resistant cells]. [Article in Japanese]. Gan to kagaku ryoho.

[52] Nishiyama M, Takagami S, Kim R, Kirihara Y, Saeki T, Jinushi K, Niimoto M, Hattori T (1988). [Inhibition of thymidylate synthetase and antiproliferative effect by 1-hexylcarbamoyl-5-fluorouracil]. [Article in Japanese]. Gan to kagaku ryoho.

