# Supplementary material for: Acid ceramidase and its inhibitors: a de novo drug target and a new class of drugs for killing glioblastoma cancer stem cells with high efficiency
Source: Oncotarget. 2017 Nov 7;8(68):112662–74. doi: 10.18632/oncotarget.22637 (PMC5762539; doi:10.18632/oncotarget.22637)
Supplement: Supplementary file 1 [file oncotarget-08-112662-s001.pdf]

Acid ceramidase and its inhibitors: a *de novo* drug target and a new class of drugs for killing glioblastoma cancer stem cells with high efficiency

SUPPLEMENTARY MATERIALS

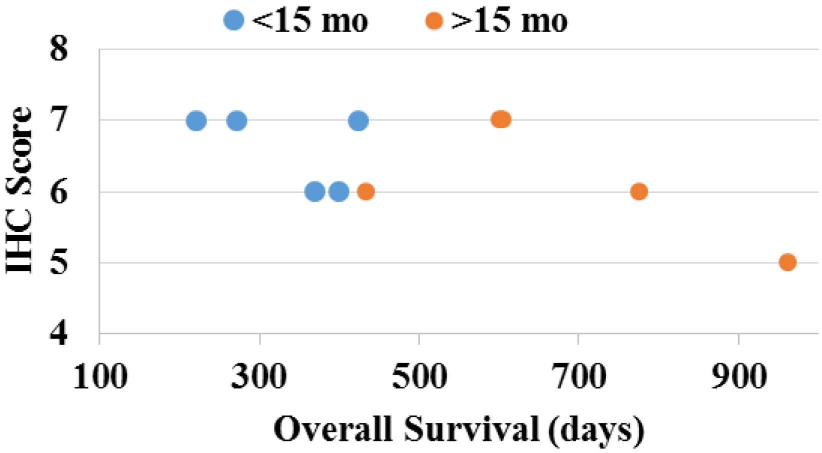

Supplementary Figure 1: Scatter plot of IHC scores vs Overall Survival in days for the nine newly diagnosed GBM patient samples stained for ASAH1. The lower IHC scores indicate longer survival, in general. The few discrepancies are possibly due to intra-tumoral heterogeneity of GBM.

Supplementary Table 1: The list of 601 biomarkers and their coefficient of determination (R^2) obtained from linear regression plots between mass spectral counts vs overall survival. See Supplementary_Table_1

Supplementary Table 2: Clinical characteristics of study subjects

Patient ID/Lane	Age (years)	Sex	Overall Survival (days)
1	25	Female	2754
2	47	Male	1385
3	65	Female	1071
4	52	Male	950
5	48	Male	614
6	39	Male	605
7	58	Male	533
8	66	Female	315
9	66	Female	291
10	64	Female	277
11	67	Female	181
12	51	Male	157
13	53	Female	105
14	65	Male	94
15	53	Male	38
